# Determinants of IL-6 levels during HIV infection

**DOI:** 10.7448/IAS.17.4.19482

**Published:** 2014-11-02

**Authors:** Álvaro H Borges, Jemma L O'Connor, Andrew N Phillips, Frederikke F Rönsholt, Sarah Pett, Michael J Vjecha, Martyn A French, Jens D Lundgren

**Affiliations:** 1 Department Infectious Diseases & Rheumatology, CHIP, Rigshospitalet, University of Copenhagen, Copenhagen, Denmark; 2 Research Department of Infection and Population, University College London, London, UK; 3 Department of Infectious Diseases, Rigshospitalet, University of Copenhagen, Copenhagen, Denmark; 4 MRC Clinical Trials Unit, UCL & Kirby Institute, University of New South Wales Australia, London, UK; 5 Veterans Affairs Medical Center, Institute for Clinical Research, Washington, DC, USA; 6 Department of Clinical Immunology, Royal Perth Hospital & PathWest Laboratory, Perth, Australia

## Abstract

**Introduction:**

Elevated IL-6 levels have been linked to increased risk of cardiovascular disease (CVD), cancer and death. Compared to the general population, treated HIV+ persons have 50–100% higher IL-6 levels, but few data on the determinants of IL-6 levels during HIV infection currently exist.

**Material and Methods:**

Participants in three international HIV trials (SMART, ESPRIT and SILCAAT) with IL-6 plasma levels measured at baseline were included (*N*=9864). Factors independently associated with log2-transformed IL-6 level were identified by multivariate linear regression; exponentiated estimates corresponding to fold differences (FDs) in IL-6 were calculated. Demographics (age, gender, race, BMI) and HIV-specific variables (nadir and entry CD4 counts, HIV-RNA, use of different ART regimens) were investigated in all three trials. In SMART (*N*=4498), smoking, comorbidities (CVD, diabetes, hepatitis B/C [HBV/HCV]), HDL-cholesterol, renal function (eGFR) and educational level were also assessed.

**Results:**

Demographics associated with higher IL-6 were older age (FD [95% CI]: 1.09 [1.08–1.11] per 10 yr) and higher BMI (1.02 [1.01–1.04] per 5 kg/m^2^), whereas black race was associated with reduced IL-6 (0.96 [0.93–0.99]). As for HIV variables, patients not receiving ART (1.36 [1.29–1.43]) and with higher HIV-RNA (1.24 [1.01–1.52] for >100,000 vs. ≤500 copies/mL) had increased IL-6. Participants taking protease inhibitors (PI) had higher IL-6 (1.14[1.09–1.19]). Higher nadir CD4 count (0.98 [0.97–0.99]/100 cells/µL) was related to lower IL-6. All evaluated comorbidities were related to higher IL-6; FDs in IL-6 were 1.08 [1.04–1.12] for smoking, 1.12 [1.02–1.24] for CVD, 1.07 [1.00–1.16] for diabetes and 1.12 [1.02–1.24] for HBV (1.15 [1.02–1.30]) and 1.53 [1.45–1.62] for HCV. IL-6 increased with decreasing eGFR (0.98 [0.97–1.00]/10 mL/min) and HDL-cholesterol (0.98 [0.96–0.99]/10 mg/mL). Lower education was related to higher IL-6 (1.09 [1.03–1.15] for high school vs. bachelor's degree).

**Conclusions:**

Higher IL-6 levels were associated with older age and non-black race, higher BMI and lower HDL-cholesterol, ongoing HIV replication, low nadir CD4 counts, comorbidities and decreased renal function. This suggests that there are multiple causes of inflammation in treated HIV infection. A possible contribution from PI use was also observed. Contribution from inflammation to explain variation in clinical outcomes for these factors should be investigated.

